# The role of biomarkers in the diagnosis and risk stratification of acute coronary syndrome

**DOI:** 10.4155/fsoa-2017-0036

**Published:** 2017-10-27

**Authors:** Sanoj Chacko, Sohaib Haseeb, Benedict M Glover, David Wallbridge, Alan Harper

**Affiliations:** 1 KeeleCardiovascular Medicine, Keele University, Keele, Staffordshire, ST5 BG, UK; 2 Heart Rhythm Service, Kingston General Hospital, Queen's University, Kingston, ON, Canada

**Keywords:** acute coronary syndrome, biomarkers, creatine kinase, heart-type fatty acid-binding protein, myeloperoxidase, troponin

## Abstract

Coronary artery disease is a growing concern. Although traditional biomarkers, such as troponins and creatine kinase, play a central role in the diagnosis, risk stratification and management of coronary artery disease, they are unable to detect myocardial ischemia in the absence of necrosis. Therefore, early detection of ischemia in patients presenting with acute coronary syndrome still remains a burning question. High-sensitivity troponin is evolving as a reliable biomarker in this regard and has been absorbed into clinical practice. Biomarkers are currently the focus of immense interest as it not only helps with diagnosis and management but also helps to understand the pathophysiology of the disease process. In addition, analysis using a multimarker strategy has also proven to be a very useful tool in risk stratification. This review will focus on the biomarkers and its application in the diagnosis and risk stratification of acute coronary syndrome.

## Lay abstract

Cardiovascular diseases are a common group of diseases that affect a large population and account for a significant number of deaths worldwide. Acute coronary syndrome (ACS) is a growing public health concern, where inadequate blood flow to the heart muscle results in reversible or irreversible damage to the cardiomyocyte. Early detection of ACS is crucial to deliver appropriate timely therapy to prevent myocardial necrosis and heart failure. Biomarkers are measurable and quantifiable biological substances that are used as indicators in the early diagnosis and management of patients with ACS. This article provides an overview of this topic and provides suggestions for the future.

Cardiovascular disease is a very common diagnosis and a leading cause of death in both men and women. It accounts for 30% of deaths worldwide, including 40% in high-income countries and approximately 28% in the developing nations [1]. Data from the Framingham study, a 44-year follow-up of cohort, suggest that the incidence of coronary events significantly increases beyond the age of 65, from 33 to 65% in men, and from 28 to 58% in women [2]. In 2007, the European Society of Cardiology, American College of Cardiology Foundation, American Heart Association and World Heart Federation task forces classified myocardial infarction (MI) as follows: Type 1 = spontaneous MI as a result of primary coronary event such as plaque rupture; Type 2 = MI due to increased oxygen demand or reduced supply such as coronary spasm, embolism and arrhythmia; Type 3 = sudden unexpected cardiac death secondary to presumed MI; Type 4 = MI associated with percutaneous coronary intervention (PCI); and Type 5 = MI associated with coronary artery bypass surgery. Biomarkers play a key role in the diagnosis of acute coronary syndrome (ACS); however, it is a marker of myocardial necrosis. From the onset of ischemia, it could take as least as 20 min for myocardial necrosis to develop as previously demonstrated in the animal models, but for complete necrosis of all myocardial cells involving the culprit epicardial coronary vessel, it could take long as 2–4 h depending on other factors such as presence of collaterals, persistent or intermittent coronary occlusion and ischemia preconditioning [3]. The diagnosis of ACS has traditionally depended upon a combination of ischemic symptoms, ECG changes and elevation in serum biomarkers. However, the symptoms are often rather atypical or absent, and around 33% of the patients arriving at the emergency department with MI may not have chest pains [4]. Similarly, ECG changes that aid with early diagnosis may be nonspecific or even absent in around 40% of the patients [5]. Moreover, ST-segment changes are also observed in other cardiac conditions like pericarditis, left ventricular hypertrophy, cardiomyopathies and channelopathies, which can add to the diagnostic dilemma.

Despite improved laboratory assays for cardiac-specific biomarkers and a revised definition for ACS, early detection of coronary ischemia in unselected patients with chest pain remains a major challenge [6]. Although conventional biomarkers play a significant role in diagnosing ACS, it is still insufficient in picking up those high-risk patients in the early phase of myocardial ischemia, which results in a delayed treatment plan [7]. Thus, growing interest in the identification of novel biomarkers of myocardial ischemia has spiked in recent years.

## Biomarkers

Biomarker, a term introduced in 1989 as a measurable and quantifiable biological substance used as an indicator of a specific biologic state relevant to a specific disease process [8]. In 2001, the ‘Biomarker Definitions Working Group’ standardized the definition of a biomarker “a characteristic that is objectively measured and evaluated as an indicator of normal biologic process, pathogenic process or pharmacologic responses to therapeutic intervention” [9]. Biomarkers play an important and indisputable role in the diagnosis and management of patients with ACS. They also provide information regarding the pathophysiology, and are useful in improving treatment strategies for patient care. Specific biomarkers reflect different components of the pathophysiology of ACS: troponins are markers for myocardial necrosis, C-reactive protein and myeloperoxidase (MPO) reflect inflammatory process, and natriuretic peptides indicate neurohormonal activation and hemodynamic stress. The emergence of  cardiac biomarkers has not only provided a unique insight into the disease process, but also represent an essential criterion in the definition of ACS [10].

**Figure 1.**
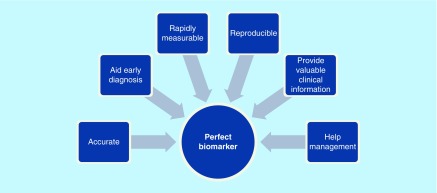
**Represents the benchmarks of a perfect biomarker.**

**Figure 2.**
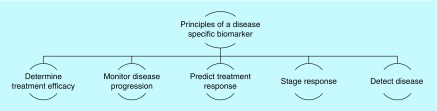
**The fundamental principles of a disease-specific biomarker.**

There have been considerable advances over the years that have led to a comprehensive understanding of the pathophysiology and bimolecular basis of coronary artery disease (CAD). As a consequence, novel cardiac biomarkers are an exciting and fascinating area of research. Novel technologies have allowed us to screen large samples of blood in a much reduced time than ever before. This provides a larger scope for researchers to apply their understanding according to the ever-growing clinical demand. There has been rapid growth in the number of novel biomarkers that emphasizes the importance of their evaluation [11]. An ideal biomarker should convincingly demonstrate its value and utility in helping with diagnosis, disease stratification and prognostication, beyond that of the existing markers. Benchmarks of a perfect biomarker and the fundamental principles of a disease-specific biomarker are shown in Figures 1 & 2, respectively. So far, the largest wealth of knowledge lies with troponins, MB isoenzyme of creatine kinase (CK-MB), myoglobin and lactate dehydrogenase. However, many other potential biomarkers have emerged and are under intense research.

## Biomarkers in the diagnosis & risk stratification of ACS

ACS, a broad term used in clinical practice, represents three primary presentations that form a part of the continuum of ACS. Depending upon patients’ cardiac symptoms, ECG changes and biomarker response, the definition is classified as: ST elevation myocardial infarction: an ST-segment elevation in the ECG with typical cardiac chest pain and rise in cardiac enzymes; non-ST elevation myocardial infarction (NSTEMI): presentation of cardiac chest pain with raised cardiac enzymes in the absence of an ST-segment elevation, but may have either ST depression, T inversion or equivocal changes; unstable angina (UA): presentation of cardiac chest pain, at rest, in the absence of any enzymatic rise, but a high risk of developing a future heart attack. Cardiac biomarkers play a major role in the diagnosis of ACS, particularly in situations where other diagnostic evidences are lacking, such as atypical cardiac chest pain or nonspecific ECG changes. Several biomarkers have emerged as a useful diagnostic tool in ACS, such as troponin, CK, CK-MB, LDH and myoglobin. By definition, elevation of one or more of the above markers is seen in all ACS patients [12], at various time courses (Table 1). However, because of their increased specificity compared with other markers, serum troponins are currently the preferred biomarker for the diagnosis of a myocardial injury.

**Table 1.** **Time course of biomarker response in myocardial infarction.**

**Markers**	**Onset**	**Peak**	**Duration**
Troponin	3–12 h	18–24 h	10 days

Creatine kinase (total and MB)	3–12 h	18–24 h	36–48 h

Lactate dehydrogenase	6–12 h	24–48 h	6–8 days

Myoglobin	1–4 h	6–7 h	24 h

## Troponins

Cardiac troponins I and T are proteins that control the calcium-mediated interaction of actin and myosin. Troponins are currently the golden standard test for the diagnosis of ACS. They are elevated at 3–6 h after the onset of symptoms, and remain elevated for up to 10 days. This not only helps with diagnostic precision, but also permits late diagnosis [13]. According to the European Society of Cardiology and American College of Cardiology consensus recommendations, troponin concentration above 99th percentile of normal is considered the diagnostic criteria for MI [14,15] and most studies support this diagnostic criterion [16]. Troponin has a good specificity and sensitivity for detecting myocardial necrosis and has also emerged as a powerful predictor of prognosis [17]. The correlation of the degree of troponin elevation with mortality has been shown in major trials like the Thrombolysis in Myocardial Infarction IIIB trial [18], in which troponin levels were measured at baseline in 1404 patients with UA or NSTEMI. The authors of this trial reported that the degree of troponin rise correlated with 42-day mortality, and moreover, a rise in troponin levels correlated with a progressive increase in risk of mortality. Similar findings were also noted in the Global Utilization of Streptokinase and t-PA for Occluded Coronary Arteries [19]. The Fragmin During Instability in Coronary-Artery Disease trial studied 917 patients with NSTEMI and showed that elevated troponin, measured within 24 h of presentation was associated with an increased incidence of death at a mean follow-up of 37 months [13,20].

Cardiac troponins can play an important role in primary prevention for cardiovascular event. The BiomarCaRE consortium by Blankenberg *et al*. was an investigation undertaken to evaluate troponin I concentration distributions across the content of Europe for risk stratification of cardiovascular and noncardiovascular mortality. The study cohort consisted of ten population-based studies, which encompassed 74,738 subjects with measurements for troponin I concentrations. Investigators noted that with troponin concentrations above 6 ng/l, an improved risk prediction was observed with a hazard ratio of 1.87 (95% CI: 1.72–2.03; p < 0.001) for cases, and a hazard ratio of 0.013 (95% CI: 0.011–0.016) for noncases. Troponin I concentrations in healthy subjects were associated with fatal cardiovascular events, overall mortality and cardiovascular disease. After an extensive analysis of studies, the authors concluded that an improvement of prediction of risk for cardiovascular death and overall mortality occurs with the addition of troponin I concentrations [21].

In addition to its diagnostic and prognostic usefulness, troponins are also used as a guide for selecting patients who may benefit from specific treatment strategies, including but not limited to low molecular weight heparin and cardiac catheterization. Despite troponin's obvious strengths and its significance in the management of ACS, it has some limitations: a concerning issue is that troponin concentration above the 99th percentile to the normal is used as the cut-off point for the diagnosis of MI; 99th percentile of troponin T is about 0.01 μg/l, while most routine laboratory assays do not reliably measure levels below 0.03 μg/l. Therefore, patients with troponin concentration between 0.01 and 0.03 μg/l, who will currently be classified as possible ACS according to the present criteria, are potentially missed and categorized as low risk [22]. Another point in question is the analytical techniques that involve immunoassays as opposed to the enzymatic methods used for creatine kinase. There are variations in the sensitivity and specificity of various immunoassays due to the lack of standardization and variations in antibody cross-reactivities to the various detectable forms of troponins [23,24]. Troponins are elevated in the range of 3–12 h after a myocardial injury, and detectable concentration is an indication of an irreversible myocardial injury, suggesting a delay relative to the onset of ischemia [25]. Troponins are unable to detect myocardial ischemia in the absence of necrosis, and hence unable to yield early diagnosis. Although troponin provides improved specificity for the detection of myocardial injury, its elevation does not necessarily imply the onset of ACS. Troponin elevation occurs in a variety of other clinical conditions including a moderate-to-severe pulmonary embolism with an acute right heart overload, heart failure and myopericarditis [26]. However, troponin elevations are usually modest in these disorders when contrasted to the more prolonged elevation that occurs with acute myocardial injury [27]. The risk assessment of cardiac biomarkers in patients with renal failure and normal renal function is not equivalent; therefore, the significance of troponin elevation in the setting of end-stage renal disease is controversial [28]. Although several studies in the past have reported that troponin rise in patients with chronic renal failure is an independent predictor of adverse outcome, it is possible to have an elevated troponin in the absence of true cardiac injury, due to possible re-expression of cardiac troponin in uremic myopathic skeletal muscles and due to reduced clearance.

## Higher sensitivity troponin

The older assays are referred to as ‘conventional’ and the newer assays as ‘high sensitivity’. As the name stands, the older assays are less sensitive than the newer ones. According to the 2012 expert consensus, to be classified as a high-sensitivity assay, it should have a coefficient of variance of <10% at the 99th percentile value, and a concentration below the 99th percentile should be detectable above the assay's limit of detection for >50% of healthy individuals in the population of interest [29]. High-sensitivity troponin assays detect concentrations that are tenfold lower than those detected with the standard assay, and these have shown to be independently associated with adverse cardiac outcome in patients with heart failure and stable CAD [30,31]. In a community-based prospective study of 1499 individuals, Xiao *et al*. showed that detectable troponin levels were noted in 820 participants of which most of them were older male with history of hypertension, diabetes, impaired renal function and was associated with increased subsequent risk of all-cause mortality and cardiovascular events [32]. A recent study further evaluated the association between high-sensitive troponin and 5-year outcome among patients with diabetes mellitus and stable CAD. The study showed a strong and consistent association between baseline concentrations of circulating troponins and the risk of all-cause mortality, ACS, stroke and heart failure [33]. These studies suggest that, in addition to the diagnostic role of troponin as a marker of ACS, it can also assess ongoing myocardial injury in stable patients and even seemingly healthy populations and could be employed as a reliable tool for risk stratification. Troponin's improved sensitivity has allowed us to detect its first low-level elevations, which are detectable within 90–180 min of an indexed cardiac event [34,35].

Despite the increase in accuracy and early detection of ACS with a high-sensitivity assay, there are still a few concerns. Research has shown a nominal level of high-sensitivity troponin at baseline in apparently healthy subjects, and the presence of biological variability over time. These changes could be due to circadian rhythm, seasonal changes or a random biological fluctuation around an inherent set point, specific to an individual [4,36]. Due to the variability with time and detectable levels in a healthy state, further investigations are needed to apply this in a more reliable manner.

As the sensitivity and precision of troponin assay improves, false positives have become less of a problem. However, given that there is a large differential diagnosis generated by positive troponins, positive results should be closely correlated with clinical presentations. Although troponin is a strong prognostic marker for cardiovascular death and recurrent ischemic events, further investigations are needed to provide thresholds to guide therapeutic strategies. With recent advances in technology, high-sensitivity troponins have emerged as an important biomarker with an added advantage in early diagnosis and risk stratification for patients with ACS.

## Creatine kinase

The CK-MB was the preferred marker of cardiac injury for several years. It has a high specificity for cardiac tissue, particularly in patients with ischemic symptoms in the absence of skeletal muscle damage. Increases in CK usually begin 4–6 h after the onset of MI, and peaks at around 18–24 h, while returning to baseline levels by 36–48 h [37]. A total CK elevation of twofold above normal, with a simultaneously elevated CK-MB is required for the diagnosis of an MI. However, the important drawback of this is a false positive result, which can be due to skeletal muscle damage from trauma, cardiopulmonary resuscitation, defibrillation, cardiac and noncardiac surgical procedures, cocaine abuse, intramuscular injections, convulsions, alcohol intoxication, hypothyroidism, renal failure and pulmonary embolism [38,39]. In the diagnosis of ACS, CK is comparatively less sensitive and specific than troponin. Several trials in the past have compared troponin with CK-MB. A review of around 30,000 patients in the multicenter, ‘Can Rapid Risk Stratification of Unstable Angina Patients Suppress Adverse Outcomes with Early Implementation of the American College of Cardiology (ACC)/Americal Heart Association (AHA) (CRUSADE)’ trial, showed that troponin was more sensitive and has an enhanced prognostic value than CK-MB [40]. Similarly, in a report of over 10,000 patients with ACS from the multicenter GRACE registry showed that in-hospital mortality was highest when both troponin and CK-MB were positive (7.7%), intermediate in troponin-positive/CK-MB-negative patients (3.9%) and lowest in patients in whom both markers were negative, and those who were troponin-negative/CK-MB-positive (1.7 and 2.3%, respectively) [41]. The CARMAGUE study assessed European (EU) and North American (NA) guidelines for cardiac biomarkers using a web-based questionnaire. Compilation of 533 hospitals (422 EU institutions; 91 NA institutions) revealed that cardiac troponins were the preferred biomarkers (99.5 EU; 98.7 NA) with the majority of healthcare institutions using it as a first-line biomarker. Differing laboratory practices and biomarker availability lead to differences in the use of cardiac biomarkers [42], but with the evidence from this study, it could be argued that creatine kinase, LDH and myoglobin are less important than cardiac troponins.

## Heart-type fatty acid-binding protein

Heart-type fatty acid-binding protein (H-FABP) is a small cytosolic protein that is concentrated in the cardiomyocyte. It transports fatty acids in the myocytes and is released in the circulation in response to ischemic insult. Several studies have shown that H-FABP is a sensitive marker of ACS [43]. Seino *et al*. measured H-FABP levels in 371 consecutive patients with acute chest pain and suspected MI [44]. There were 68 patients who presented within 2 h of symptoms. The sensitivity of MI at 2 h was significantly higher with serum H-FABP compared with cardiac troponin T or myoglobin (89 vs 22 and 38%, respectively). However, cardiac troponin T had a significantly higher specificity (94 vs 52%). H-FABP has shown to provide valuable information in the multimarker approach in ACS, and when measured in combination with troponins and BNP, H-FABP has proven to be very useful in identifying patients who are at an increased risk of a worsening heart failure, death and rehospitalization. Moreover, it has also shown to be useful in estimating the size of the infarct. H-FABP appeared to provide incremental information beyond the established biomarkers; however, this has not been compared and established [45], and there are no studies which directly compare H-FABP with troponins, although its smaller size, rapid release and clearance offer some theoretical advantage over troponins [46]. H-FABP appears to be a very promising biomarker of myocardial injury; however, more studies are required to support these preliminary findings.

## Myeloperoxidase

MPO is a hemoprotein mainly released by activated neutrophils, monocytes and tissue macrophages. It is emerging as a new biomarker of inflammation and has been proposed as a useful diagnostic tool in patients admitted with ACS [47]. MPO appears to play an important role in atherogenesis by its involvement in low-density lipoprotein oxidation. Recent studies have suggested an association between plasma levels of MPO and coronary atherosclerosis, and this enzyme is also found to be abundant in ruptured plaques [48]. MPO is also emerging as a useful tool in primary prevention to assess the risk for future coronary event; however, only few important studies have examined the role of MPO as a marker of risk for CAD. In relation to this, Zhang *et al*. showed that the serum MPO activity was higher in patients with CAD when compared with angiographically verified normal subjects [49]. MPO has also been very useful in the setting of ACS where high-circulating MPO levels were detected in patients with UA and acute MI when compared with patients with stable CAD, suggesting that MPO levels are a marker of instability. Its potential usefulness in risk stratification in ACS was examined in the CAPTURE trial [50]. CAPTURE trial enrolled 1265 patients with ACS. At baseline, MPO was detectable in the serum samples of all subjects, with a median of 287 μg/l. However, when compared with the low levels (<222 μg/l), MPO levels of >350 μg/l was associated with significant event rates at 72 h, 30 days and 6 months’ follow-up. There are also studies looking at MPO's long-term outcome in patients with ACS, Mocatta *et al*. studied 512 patients with acute MI and found a significant association of raised MPO levels and repeat cardiac events [51]. MPO is a marker of inflammation and oxidative stress that is emerging as a potential biomarker of ACS; however, there are only few strong quantitative analyses available; therefore, more studies are required to determine the precise role of MPO in ACS.

## Conclusion & future perspective

The traditional biomarkers currently available, such as troponins and CK have played a major role in the diagnosis, risk stratification and management of ACS. Extensive research has led to the discovery and development of potential novel biomarkers such as MPO, H-FABP and are under intense validation and research. With the emergence of novel markers, complimentary analysis using a multimarker strategy has also proven to be a very useful tool in risk stratification.

The number of novel biomarkers are expected to grow over the years to come, with the ultimate goal being the identification of markers which could potentially add more value and provide additional information to improve patient care. With the omics approach growing immensely, clinicians and researchers can use metabolomics to study these processes at the molecular level, further aiding in optimized therapies to be translated to clinical practice.

**Executive summary**

- Biomarkers are a characteristic that is objectively measured and evaluated as an indicator of normal biologic process, pathogenic process or pharmacologic responses to therapeutic intervention.
- Acute coronary syndrome (ACS) is a broad term used in clinical practice, representing three primary presentations that form a part of the continuum of ACS (ST elevation myocardial infarction, non-ST elevation myocardial infarction, angina).
- Over the years, many biomarkers have emerged as a useful diagnostic tool in ACS, most importantly troponins, and to a lesser degree CK, MB isoenzyme of creatine kinase.
- High-sensitivity troponins are currently the gold standard test for the diagnosis and management of ACS.
- Novel biomarkers are emerging as an area of immense interest in the diagnosis and risk stratification of ACS.
- Complimentary analysis using multibiomarker strategy has also proven to be a useful risk stratification tool.
